# Association of Negative Symptoms of Schizophrenia Assessed by the BNSS and SNS Scales With Neuropsychological Performance: A Gender Effect

**DOI:** 10.3389/fpsyt.2021.797386

**Published:** 2021-12-24

**Authors:** Paweł Wójciak, Klaudia Domowicz, Marta Zabłocka, Michał Michalak, Janusz K. Rybakowski

**Affiliations:** ^1^Department of Adult Psychiatry, Poznan University of Medical Sciences, Poznan, Poland; ^2^Department of Computer Science and Statistics, Poznan University of Medical Sciences, Poznan, Poland

**Keywords:** self-evaluation of negative symptoms, negative symptoms, neurocognition, brief negative symptom scale, schizophrenia

## Abstract

**Objective:** The relationship between negative symptoms and neurocognitive performance in schizophrenia is well documented, but the mechanism of these connections remains unclear. The study aims to measure the relationship between the results on the new scales for the assessment of negative symptoms such as Brief Negative Symptom Scale (BNSS) and Self-evaluation of Negative Symptoms (SNS), and the results of some neurocognition tests. The second aim is to assess a possible gender effect on these associations.

**Methods:** The study included 80 patients (40 men, 40 women) with schizophrenia, aged 19–63 (mean 38 years), during the improvement period (total PANSS score <80, unchanged pharmacological treatment in the last 3 weeks). They were assessed using the BNSS, SNS, Personal and Social Performance (PSP) scales, and the tests for neuropsychological performance such as the Trail Making Test (TMT-A, TMT-B), Stroop Color-Word Interference Test, Verbal fluency tests (VFT), Category fluency test (CFT), and Digit Symbol Substitution Test (DSST).

**Results:** Male patients obtained higher scores than females on some PANSS and BNSS items. No gender differences were observed for the SNS scale. Female patients scored better in the PSP and CFT. In male patients, a significant positive correlation between the intensity of negative symptoms measured by the BNSS and the results of PSP with the Trail Making Test was observed. In female patients, we found a positive correlation between the results of BNSS and PSP with the Stroop Color-Word Interference Test.

**Conclusion:** The obtained results confirm the relationship between negative symptoms and neurocognition in schizophrenia patients. However, in male and female patients such association was observed for different cognitive domains. Further research is needed to explain the nature of these differences.

## Introduction

Schizophrenia is a multidimensional disorder, the disease syndrome consists of positive, negative, disorganized, and affective symptoms with varying degrees of intensity, accompanied by disorders of neurocognition and social cognition. Many studies focus on assessing the mutual relations between these domains. The relationship between negative symptoms and cognitive impairment is well documented (1), it seems to be stronger (2) than for positive (3) and affective symptoms (4). The mechanism of these connections, however, remains unclear (5), the obtained results are heterogeneous, and there are also methodological controversies.

Negative symptoms and cognitive impairment share many things in common. Their frequency, course, prognostic significance, and correlation with many aspects of daily functioning are similar (6). In most studies, the severity of both disorders negatively correlates with each other (7, 8). It should be noted, however, that there are also studies in which no correlation between negative symptoms and neurocognition was found (9, 10).

To explain these discrepancies, Harvey et al. (6) suggest the possibility of the occurrence of as many as four different models of the relationship between negative symptoms and cognitive impairment. Both disorders are a common dimension of the disease, both disorders are a separate dimension but with a similar etiology, each disorder has a separate etiology but with some common elements, and finally both disorders are a separate dimension and have a separate etiology. Summing up, the authors support the conclusion that both symptoms are separate, a similar position is taken by Yolland et al. (11). In their work, they did not observe the relationship between the severity of negative symptoms and cognitive impairment, which led them to suggest that negative symptoms and neurocognition are separate constructs and require separate therapeutic strategies.

Despite the above reservations, it seems that the mutual relationship between these two symptoms is more complex and multidimensional. Harvey et al. (6) indicate that cognitive disorders appear even before the clinical onset of psychosis and, similarly to negative symptoms, can be referred to as “early symptoms,” which allows treating them as a neurodevelopmental component of schizophrenia. Ventura et al. (7) point out that negative symptoms may mediate between cognitive impairment and functional outcome, for Velligan et al. (12) negative symptoms are a behavioral consequence of cognitive impairment.

Harvey et al. (6) also indicate that the relationship between negative symptoms and cognitive impairment depends on the definition of negative symptoms while emphasizing that negative symptoms are defined based on the clinical picture, and cognitive disorders based on the tests performed. Additionally, the definition of negative symptoms differs depending on whether we are dealing with primary or secondary negative symptoms. Thus, patients with stable negative symptoms forming the deficit syndrome are characterized by a significant intensification of cognitive disorders (13).

As indicated by Milev et al. (2), the type of research tool used has a significant impact on the obtained results. In the research so far, the most frequently used scale is the Positive and Negative Syndrome Scale (PANSS). Some symptoms of cognitive nature (deficits in abstract thinking, stereotyped thinking, poor attention) are defined as negative or as general symptoms of schizophrenia by the PANSS. SANS also describes behavioral disorders such as deficits in social, occupational, and educational activity as negative symptoms, the same problem applies to attention disorders treated as negative symptoms (6). In a large systematic review of studies assessing the relationship of schizophrenia symptoms, including negative ones, with cognitive impairment, the authors cited the results of 18 studies from 2008-2019, none of the research protocols included the so-called 2nd generation scales for the assessment of negative symptoms, taking into account the NIMH- MATRICS consensus statement on negative symptoms (14, 15).

The nature and structure of the scales used so far lead, as noted by Alpert et al. (16) to evaluate individual items of negative symptoms through the prism of the global assessment of the clinical picture, which significantly affects the obtained results.

It also seems important to consider sex differences in research protocols that affect the age of onset of the disease, premorbid adjustment, course, and expression of clinical symptoms, which is particularly important in the context of the assessment of cognitive disorders and their relationship with negative symptoms (17).

Significant gender effects have been observed in schizophrenia for both negative symptoms and neurocognition. Moriarty et al. (18) suggested that negative symptom severity is greater in male patients. This was confirmed in subsequent research of Galderisi et al. (19) as well as our recent study as to the assessment on the BNSS scale (20).

The results of studies assessing the relationship between gender and cognitive processes in patients with schizophrenia are inconclusive. Some studies have reported superior cognitive function in women, others found the opposite or no gender difference, while some observed a reversal of normal sexual dimorphism (21). In numerous studies, men with schizophrenia performed worse in terms of cognitive functions, both in comparison with the control group (22) and female patients (23, 24). Only a few studies have shown better neurocognitive performance in men compared to women with schizophrenia (25, 26). There are also studies showing no difference in terms of neurocognition between men and women with schizophrenia (27, 28). As a factor assisting better cognitive performance in women with schizophrenia, estrogen and its neuroprotective role in the body are most often indicated (17, 29).

It seems that the endocrine system and its influence on the central nervous system are more closely related to the development of schizophrenia and sexual differences in the course of this disease than previously thought (30). Recent studies show that structural plasticity of the brain is regulated by hormones (31), not only in the systemic aspect, but also in the local–the brain is also capable of locally generating estrogens, either from androgens and possibly also directly from cholesterol (32, 33). The estrogen hypothesis is supported by late-onset age and second incidence peak around menopausal age in women. Indeed, estrogen deficiency is highly related to the severity of psychiatric symptoms in women during menopause (34). For example, female schizophrenia patients often have more severe symptoms in the low estrogen phase of their menstrual cycle (35). Interestingly, the negative correlation between plasma estrogen levels and schizophrenia symptoms was also reported in male patients (36). The biochemical nature of the neuroprotective effects of estrogens has not been fully identified yet, but a number of studies points to a direct implication of the dopaminergic system, in addition to glutamate and GABA (37). When it comes to testosterone, studies found that low levels of testosterone appear to be associated with more severe symptoms, although results are less consistent than for estrogens (38). For instance, studies found that schizophrenia patients with low levels of testosterone often have predominantly negative symptoms, and serum testosterone levels are associated with greater severity of negative symptoms (39). Another important hormone associated with the pathogenesis of schizophrenia appears to be oxytocin. Several studies suggest that schizophrenia patients with higher levels of plasma oxytocin develop fewer psychotic symptoms (40), and have better cognition (41). Oxytocin is thought to regulate central dopamine, and might therefore exhibit antipsychotic effects (42).

The study aims to evaluate the relationship between negative symptoms assessed by the so-called 2nd generation scales such as the Brief Negative Symptoms Scale (BNSS) (43), and the Self-evaluation of Negative Symptoms (SNS) (44), with neuropsychological performance, in patients with chronic schizophrenia. The hypothesis was put forward postulating that such a relationship can be connected with gender.

## Methods

### Study Design and Participants

This is a cross-sectional study. The study participants were recruited among subjects attending the outpatient and inpatient unit of the Department of Adult Psychiatry, Poznan University of Medical Sciences. The recruitment was carried out from October 31, 2016, to July 15, 2017.

Eighty patients (40 male, 40 female), aged 19–63 (mean 38 ± 11) years, with a diagnosis of schizophrenia, according to ICD-10 and DSM-5 were included. Their onset of illness was 26 ± 8 years, the duration of illness was 12 ± 9 years, and the number of hospitalization was 6 ± 6. They were either inpatients (60 subjects) or were under the care of the outpatient clinic (20 subjects), at the Department of Adult Psychiatry, Poznan University of Medical Sciences. All patients remained in the phase of symptomatic stabilization defined as achieving a total PANSS score of the maximum of 80 points. They received unchanged pharmacological treatment in the last 3 weeks (inpatients) or 3 months (outpatients). Four male and four female patients were treated with haloperidol. The remaining subjects received atypical antipsychotics (olanzapine, clozapine, aripiprazole, risperidone, quetiapine, amisulpride, and ziprasidone) the proportion of which was comparable in male and female patients.

Participants with significant physical, visual, verbal impairments, neurological disorders, and substance abuse or dependence, were excluded.

The local Ethics Committee approved the study (Agreement no 1122/16), and it was performed in accordance with the ethical standards of the Declaration of Helsinki (45). All participants signed a written informed consent to participate in the study, after receiving a detailed explanation of the study's procedures and goals.

### Neuropsychological and Clinical Assessment

#### General Psychopathological Assessment

The Positive and Negative Syndrome Scale (PANSS) PANSS was developed by Kay et al. (46). It is a 30-item rating scale developed specifically to assess patients with schizophrenia. It is divided into three subscales, a positive scale with seven positive symptoms (P1-P7), a negative scale with seven negative symptoms (N1-N7), and a general psychopathology scale with 16 items (G1G16). Psychopathology severity is defined as 1 = absent; 2 = minimal; 3 = mild; 4 =moderate; 5 = moderate-severe; 6 = severe; 7 = extreme.

#### Assessment of Negative Symptoms

##### The Brief Negative Symptoms Scale

The BNSS was developed by Kirkpatrick et al. (43). The scale defines negative symptoms as the absence or decrease in behaviors and subjective experiences that are normally present in a person from the same culture and age group. The scale has 13 items organized into six subscales: anhedonia (intensity and frequency of pleasure, the intensity of expected pleasure), distress (subject's experience of unpleasant or distressing emotion of any kind, such as sadness, depression, anxiety, grief, anger), asociality (reported as reduced social activity accompanied by decreased interest in forming close relationships with others–behavior, internal experience), avolition (reported as a reduction in the initiation of and persistence in activity–behavior, internal experience), blunted affect (which refers to a decrease in the outward expression of emotion–facial expression, vocal expression, gestures), and alogia (reported as poverty of speech–the quantity of speech, spontaneous elaboration of speech). The examination is of an interview nature, based on a manual including, among other things, prompts and suggested questions. All items are rated on a 7-point scale (0–6), with anchor points ranging from a symptom being absent (0) to severe (6). The time frame for the ratings is 1 week. The basis for the interview is provided by the patient–participant observation also constitutes an important element–or, if needed, data obtained from external sources. The Polish version of BNSS was used in the study (47).

##### The Self-Evaluation of Negative Symptoms

The SNS was developed by Dollfus et al. (44). The scale evaluates all five groups of negative symptoms, i.e., social withdrawal, blunted affect, avolition, anhedonia, and alogia. Social withdrawal (asociality) assesses social relationships as well as the patient's propensity to establish new relationships; diminished emotional range (blunted affect) reflects a presence of happiness or sadness in situations in which they are usually felt; avolition relates to motivation, energy level and the ability to achieve goals; anhedonia describes the experienced and expected pleasure; alogia (poverty of speech) is evaluated by the subjective assessment of the examined person. The scale contains 20 items, four items for each negative symptom, and the evaluation pertains to the previous week. For maximal simplification, the number of responses was limited to 3: “strongly agree” scoring 2, “somewhat agree” scoring 1, and “strongly disagree” scoring 0. The total score is the sum of the 20 items, ranging from 0 (no negative symptoms) to 40 (severe negative symptoms). The Polish version of SNS was used in the study (48).

#### Assessment of Social Functioning

The Personal and Social Performance scale (PSP) evolved based on the social functioning component of the DSM-IV Social and Occupational Functioning Assessment Scale (SOFAS) as an effort to assess social functioning in schizophrenia. It is being proposed as an improvement over the Global Assessment of Functioning (GAF) scale and SOFAS. PSP is a 100-item scale, divided into 10 similar intervals. The score is based on the assessment of patient's performance in four categories: socially useful activities, personal and social relationships, self-care, disturbing and aggressive behavior. PSP provides a score between 1 and 100, divided into 10 equal intervals to rate the degree of difficulty. Higher scores represent better personal and social functioning (49).

#### Assessment of Neurocognition

##### Trail Making Test (TMT-A, TMT-B)

Trail Making Test is used to assess working memory and as an indicator of visual scanning, graphomotor speed, and executive function. In part A, the subject is asked to connect the circles marked with numbers from 1 to 25 as quickly as possible, which allows the measurement of psychomotor speed. In part B, the subject's task is to as quickly as possible connect the circles marked with numbers 1–13 and letters A-L in the order 1-A-2-B-3-C, etc. This operation requires keeping two different sequences of numbers and letters in working memory. The result of the test is the time and correctness of parts A and B of the test (50). We also calculated the TMT B-A difference as proposed in recent studies (51).

##### Stroop Color–Word Interference

Test Stroop Color–Word Interference Test is used to evaluate verbal working memory. The test consists of two parts. In the first one (Reading Color Names in Black, RCNb, Stroop A), the respondent is asked to read the words (color names) written in black on a white sheet as soon as possible. In the second part (Naming Color of Word different, NCWd, Stroop B), the respondent's task is to name the font color of individual words, whereby the font color of the word is different from the color indicated by him. This activity requires a change in the form of reaction (switching from content to color). The result of the test is the time needed to complete the first and second parts and the number of incorrect answers (52). The difference between Stroop B-A was also calculated (53).

##### Verbal Fluency Tests and Category Fluency Test

Verbal fluency tests and Category fluency test are used to evaluate speech functions, and semantic memory access and executive functions. The tests assess the ability to pronounce words fluently in accordance with a specific criterion in a given time (for 60 seconds). This criterion is a given letter (in VFT) or category (in CFT). In the Polish version used in the study, in the part assessing verbal fluency, the respondent's task is to list for 1 min as many words starting with the letters of the alphabet as possible: K, O, S, and in the part assessing categorical fluency, the greatest number of words from three categories: animals, vegetables, fruits (54, 55).

##### Digit Symbol Substitution Test

Digit Symbol Substitution Test is a wordless test on the Wechsler Adult Intelligence Scale (WAIS-R). It is used to evaluate motor speed, attention, and visuoperceptual functions. The task of the examined person is to assign symbols to digits according to a given key within a specified time (90 s). The result of the test is the number of correctly assigned symbols (56, 57).

### Statistics

Continuous data is presented as means and standard deviations. Except of the descriptive statistics the effect size was denoted (d Cohen). The Shapiro-Wilk test was applied to check if data follow the normal distribution. Because the data of BNSS, SNS, and PSP were consistent with a normal distribution, the gender differences were assessed by the two-tailed *t*-test. The data of neurocognitive tests was not normally distributed which is why the Box-Cox transformation of non-normal dependent variables was performed in order to assess a normal shape. Additionally, the Benjamini–Hochberg adjusted *p*-value for multiple comparisons was calculated. Statistical relationships between BNSS, SNS, PSP and the results of neurocognitive tests were calculated using Pearson correlation coefficient. The statistical analysis was performed with the use of TIBCO Software Inc. (2017). Statistica (data analysis software system), version 13. http://statistica.io. All test were considered significant at *p* < 0.05.

## Results

The mean age of women with schizophrenia was higher than men (41 vs. 35, *p* = 0.048), the mean number of years of education was greater in the group of women than in men (13 vs 12, *p* = 0.046). There was no gender difference in disease duration and number of hospitalizations (see Table 1).

**Table 1:** Demographic characteristics of the study group.

**Category**	**Total** ***n* = 80**	**Women** ***n* = 40**	**Men** ***n* = 40**	***p***
Age Mean ± SD (years)	38 ± 10	40 ± 12	35 ± 8	0.048^*^
Years of education Mean ± SD	12 ± 2	13 ± 2	12 ± 2	0.046^*^
Illness duration Mean ± SD (years)	12 ± 9	13 ± 9	10 ± 9	0.090
Number of hospitalizations Mean ± SD	6 ± 6	6 ± 6	6 ± 6	0.785

* *Difference between men and women significant p < 0.05*.

The comparison of the results of psychometric tests in male and female schizophrenia patients is shown in Table 2.

**Table 2:** The comparison of the results of clinical and psychometric tests in female and male schizophrenia patients.

**Parameter**	**Total**		**Women**		**Men**		**Effect size**	***p*-value**	Adjusted *p*-value^*^
	**Mean**	**SD**	**Mean**	**SD**	**Mean**	**SD**	**d Cohen**		
PANSS positive	13.6	3.1	12.8	3.1	14.3	3	−0.5	0.041	0.082
PANSS negative	16.6	5	15.1	4.9	18.2	4.8	−0.6	0.006	0.022^*^
PANSS general	29	5.3	28.2	6.2	29.7	4.3	−0.3	0.221	0.221
PANSS sum	59.2	10.8	56.2	11.7	62.2	8.8	−0.6	0.012	0.035^*^
BNSS anhedonia	6.2	3.4	5.6	3.7	6.6	3.1	−0.3	0.156	0.156
BNSS distress	1.3	1	1	1	1.7	1	−0.7	0.001	0.010^*^
BNSS asociality	3.9	2	3.3	2	4.5	1.9	−0.6	0.009	0.050^*^
BNSS avolition	4.1	2.1	3.7	2.1	4.6	2	−0.4	0.086	0.156
BNSS blunted affect	4.9	3.2	4.2	3.2	5.7	3	−0.5	0.038	0.153
BNSS alogia	2.6	2.2	2.1	2	3	2.4	−0.4	0.086	0.156
BNSS sum	23.3	11.5	20.2	11.7	26.4	11.6	−0.5	0.020	0.099
SNS social withdrawal	2.9	2	2.5	2.1	3.3	1.8	−0.4	0.076	0.455
SNS diminished emotion	3.4	2.1	3	2.1	3.7	2	−0.3	0.144	0.499
SNS alogia	3.8	2.4	3.6	2.6	4	2.3	−0.2	0.499	0.499
SNS avolition	3.8	2.3	3.5	2.5	4	2	−0.2	0.388	0.499
SNS anhedonia	2.8	2.4	2.5	2.5	3.1	2.4	−0.2	0.301	0.499
SNS sum	16.7	8.8	15.2	9.9	18.2	7.4	−0.3	0.144	0.499
PSP	60.4	14.5	68.1	14.1	60.7	14.1	0.5	0.021	0.021^*^

* *Difference between men and women significant p ≤ 0.05. Significant differences are marked in bold*.

Among the PANSS scores, there was a significantly higher score on negative symptoms and total score in male patients (both *p* < 0.05, d Cohen 0.6, medium effect size). On the BNSS scale, there was a significantly higher score of male patients in subscales of distress (*p* = 0.01, d Cohen 0.7) and asociality (*p* = 0.05, d Cohen 0.6). There was also a tendency (*p* < 0.1, d Cohen 0.5) for the higher total BNSS score. No gender differences were found as to the assessment on the SNS scale.

Female patients scored significantly better on the PSP scale (68.1 vs. 60.7, *p* = 0.021, d Cohen 0.5)

The comparison of the results of neurocognition tests in male and female schizophrenia patients is shown in Table 3.

**Table 3:** The comparison of the results of neurocognition tests in female and male schizophrenia patients.

**Parameter**	**Total**		**Women**		**Men**		**Effect size**	***p*-value**	Adjusted *p*-value^*^
	**Mean**	**SD**	**Mean**	**SD**	**Mean**	**SD**	**d Cohen**		
TMT-A	44.7	23.1	42.7	17.8	46.6	27.4	−0.2	0.457	1.000
TMT-B	113.2	73.1	108.8	51.8	117.6	89.9	−0.1	0.593	1.000
TMT B-A	68.5	60.5	66.1	45.6	71	72.3	−0.1	0.718	1.000
Stroop A time	28.6	12.6	28.6	12.1	28.6	13.1	0.0	1.000	1.000
Stroop B time	81.7	39.1	77.6	35.6	85.7	42.2	−0.2	0.360	1.000
Stroop B-A time	53.1	34.0	49.1	33.2	57.1	34.7	−0.2	0.293	1.000
Stroop B incorrect answers	4.6	12.6	4.6	16.4	4.4	7.2	0.0	0.937	1.000
Category fluency test	36.6	13.4	42.7	12.2	30.4	11.6	1.0	<0.001	**<0.001**
Verbal fluency tests	32.4	14.7	34.8	15.2	30	13.8	0.3	0.138	1.000
Digit Symbol	34.6	14.8	36.7	15.9	32.5	13.5	0.3	0.213	1.000

* *Difference between men and women significant p < 0.001. Significant differences are marked in bold*.

No significant gender differences were observed except that female patients performed significantly better on the Category Fluency Test (*p* < 0.001, d Cohen 1.0).

The relationships between the results of women and men on the BNSS, SNS, and PSP scales and the results of neurocognitive tests are shown in Table 4.

**Table 4:** Relationships between the results of men and women on the BNSS, SNS, and PSP scale and the results of neurocognition tests.

**Parameter**	**Male**				**Female**			
	**TMT-A**	**TMT-B**	**Stroop A**	**Stroop B**	**TMT-A**	**TMT-B**	**Stroop A**	**Stroop B**
BNSS anhedonia	0.399 *p* = 0.011	0.328 *p* = 0.039	0.127	0.179	0.027	−0.203	0.306	0.368 *p* = 0.019
BNSS asociality	0.343 *p* = 0.03	0.359 *p* = 0.023	0.209	0.207	−0.099	−0.250	0.380 *p* = 0.015	0.3
BNSS avolition	0.459 *p* = 0.003	0.356 *p* = 0.024	0.18	0.257	−0.053	−0.284	0.308	0.405 *p* = 0.009
BNSS blunted affect	0.524 *p* = 0.001	0.398 *p* = 0.011	−0.026	0.225	0.07	−0.255	0.24	0.509 *p* = 0.001
BNSS alogia	0.523 *p* = 0.001	0.414 *p* = 0.008	0.284	0.266	0.079	−0.216	0.24	0.585 *p* = 0.001
BNSS sum	0.502 *p* = 0.001	0.406 *p* = 0.009	0.158	0.254	0.009	−0.272	0.311	0.487 *p* = 0.001
SNS social withdrawal	0.165	0.263	0.007	0.011	−0.114	−0.113	0.432 *p* = 0.005	0.148
SNS diminished emotion	0.285	0.225	0.003	0.099	0.013	−0.166	0.33	0.35
SNS alogia	0.142	0.069	0.216	0.005	0.038	−0.153	0.337	0.129
SNS avolition	−0.715	0.208	0.067	−0.037	0.004	−0.013	0.166	0.21
SNS anhedonia	0.215	0.209	−0.175	0.001	0.026	−0.055	0.388 *p* = 0.013	0.126
SNS sum	0.218	0.275	0.029	0.019	−0.001	−0.097	0.391 *p* = 0.012	0.23
PSP sum	−0.439 *p* = 0.005	−0.343 *p* = 0.03	−0.163	−0.245	−0.001	0.124	−0.315 *p* = 0.047	−0.367 *p* = 0.02

*Pearson correlation coefficient and “p” of significant correlations were given*.

In male patients, we found a significant positive correlation between BNSS total and subscales and a significant negative correlation between the results on PSP with the Trail Making Test (TMT-A, TMT-B).

In female patients, a significant positive correlation between the intensity of negative symptoms measured by the BNSS total and most subscales, and the results of Stroop B was found. The BNSS asociality correlated with the results of Stroop A. The intensity of negative symptoms measured by the SNS sum, social withdrawal, and anhedonia subscale correlated with the results of Stroop A. A significant negative correlation between the results of PSP with the results of both Stroop A and B was observed.

## Discussion

The results obtained may indicate gender differences in schizophrenia patients in the assessment of negative symptoms and personal and social performance as well as in the correlations between negative symptoms and the results of neurocognitive tests. In male patients, the higher score on BNSS and worse results on PSP make a corroboration of our previous work (58). Also, the better results in schizophrenic women on PSP were recently confirmed by Spanish investigators (59).

The main aim of our study was to investigate the correlation between negative symptoms and neurocognitive functions. Such correlation of negative symptoms with various indexes of neurocognition has been described in many papers (5, 6, 60). In all these studies, the negative symptoms were assessed by the PANSS. However, in a recent validation of the BNSS in Korean patients with schizophrenia, a correlation of negative symptoms assessed by this scale with neurocognitive tests of verbal memory, processing speed, and attention was found (61).

Recently, Eack and Keshavan (62) suggested three potential underpinnings of such correlation. First, that cognitive and negative symptoms share similar underlying pathophysiology giving rise to their overlap and impact on functioning. Second, that cognitive impairment leads to greater negative symptoms and reduced functional performance, and third, that negative symptoms lead to impaired cognitive abilities that drive poor functional outcomes. In this respect, is of importance the paper of Luther et al. (63) in which in the 20-year longitudinal study, it was found that greater negative symptoms predicted reduced neurocognition and indirectly–impaired work functioning.

In our research, the results of the neurocognitive tests were numerically worse in males, however, the only significant difference was in the category fluency. Whereas, significant correlations between negative symptoms and the results on some neurocognitive tests were obtained. However, in male and female patients they were observed for different cognitive tests. In males, a significant positive correlation between the intensity of negative symptoms measured by the BNSS and the Trail Making Test was observed. In female patients, we found positive correlations between the results of the BNSS and Stroop Color-Word Interference Test. Interestingly, similar correlations in male and female schizophrenia patients were obtained between these neurocognitive tests and the results of the PSP scale.

Therefore, the most intriguing result of our study is a relationship obtained in male and female schizophrenia patients between negative symptoms and social functioning, with different cognitive domains. In men, increased negative symptoms and worse functioning were correlated with inferior results of TMT test measuring visual attention and task switching, while in female patients such correlations were obtained with a worse outcome of the Stroop test, assessing the ability to inhibit cognitive interference. Probably, it can be speculated that these clinical and functioning disturbances are mostly underpinned by different cognitive factors depending on gender.

Why impaired cognitive abilities involved in the Trail Making Test are related to the intensity of negative symptoms and social performance in men but not in women? It can be speculated that in men, impaired psychomotor speed, attention, and mental flexibility are more important than in women for a generation of negative symptoms and social functioning. A similar question can be asked, why impaired performance on the Stroop test is related to the intensity of negative symptoms and social performance in women but not in men? Probably, in women, impaired ability to inhibit cognitive interference is more important than in men for a generation of negative symptoms and social functioning.

Regardless of the neurophysiological mechanisms of these differences, the results may point to distinctive features of negative symptoms in male and female schizophrenia patients. This was also shown in our recent study in which in female patients, a significant positive correlation between the intensity of negative symptoms measured by the BNSS scale and the concentration of high-density lipoprotein (HDL) cholesterol, and a trend for negative correlation with BMI was observed. Whereas, such correlations were not found in male patients (58).

The study suffers from four major limitations. First, the experimental sample consisted of chronic and clinically stable subjects with schizophrenia and did not include a control group (e.g., healthy controls). Second, we did not include the measure of IQ, which is important for cognitive functioning in schizophrenia and potential differences between groups. Third, the study took place in a controlled environment, which resulted in a low ecological validity of the used neuropsychological assessment, consequently, it is more difficult to generalize the study's findings. Last, but not least, the chlorpromazine equivalents of drugs that were administered to patients and which may have an important role for cognitive functioning in schizophrenia were not recorded in the study. Given the importance of the above limitations, future research should take them into account.

In conclusion, the results obtained confirm the relationship between negative symptoms and neurocognition in schizophrenia patients, although for different cognitive domains, depending on gender. Considering the above aspects together seems to be important from a clinical point of view, as suggested by Li et al. (34). Consideration of gender differences in schizophrenia provides an important insight for understanding the sex-specific characteristics of the disease's onset, symptoms, and the opportunity to deliver sex-specific treatments and care for schizophrenia. The above observations have already been reflected in previous studies in which an integrated therapeutic approach with the combination of neurocognition and social cognition might be a more effective approach to treating the symptomatology of people with schizophrenia (1, 64).

Further research is needed to confirm these findings and to explain the nature of these differences. It seems particularly important to correctly define negative symptoms, to standardize tools for the assessment of neurocognition, and in the aspect of gender differences-to take into account such differentiating factors as the endocrine system or molecular-genetic findings. Study design should also take into account ecological validity in subsequent studies, to predict better behaviors in real-world settings.

## Data Availability Statement

The original contributions presented in the study are included in the article/supplementary material, further inquiries can be directed to the corresponding author.

## Ethics Statement

The studies involving human participants were reviewed and approved by Bioethics Committee, Poznan University of Medical Sciences, Poznan, Poland. The patients/participants provided their written informed consent to participate in this study.

## Author Contributions

PW took part in the design of the study and wrote the first draft of the manuscript. KD performed psychometric assessments. MZ performed neurocognitive assessments. MM performed statistical calculation. JR elaborated the design of the study and wrote the final version of the manuscript. All authors accepted the final version of the manuscript.

## Funding

The paper was helped by own funds of the Department of Adult Psychiatry, Poznan University of Medical Sciences.

## Conflict of Interest

The authors declare that the research was conducted in the absence of any commercial or financial relationships that could be construed as a potential conflict of interest.

## Publisher's Note

All claims expressed in this article are solely those of the authors and do not necessarily represent those of their affiliated organizations, or those of the publisher, the editors and the reviewers. Any product that may be evaluated in this article, or claim that may be made by its manufacturer, is not guaranteed or endorsed by the publisher.
